# Endovenous Thermal Ablation for a Varicose Vein Patient with Factor XII Deficiency: A Case Report

**DOI:** 10.3400/avd.cr.20-00137

**Published:** 2020-12-25

**Authors:** Nozomu Shirasugi, Sadaaki Horiguchi, Takamitsu Tanaka, Hiroyuki Shirato, Hisako Ono, Kazuo Kawasugi

**Affiliations:** 1 Varicose Vein Center and Department of Vascular Surgery, Yokohama Asahi Chuo General Hospital, Yokohama, Kanagawa, Japan; 2 Varicose Vein Center, Aisei Hospital, Tokyo, Japan; 3 Department of Dermatology, Teikyo University School of Medicine, Tokyo, Japan; 4 Department of Clinical Laboratory, Aisei Hospital, Tokyo, Japan; 5 Department of Hematology, Teikyo University School of Medicine, Tokyo, Japan

**Keywords:** varicose veins, endovenous thermal ablation (ETA), surgery, factor XII (FXII) deficiency

## Abstract

Factor XII (FXII) deficiency is a rare coagulation disorder, and its potential relationship with venous thrombosis was reported. Here we present a case of a 67-year-old woman with FXII deficiency who successfully underwent endovenous thermal ablation (ETA) for primary varicose vein due to the incompetent great saphenous vein (GSV). The FXII deficiency was revealed through preoperative examinations, and the patient underwent ETA as a day surgery. For prophylaxis of thrombosis, she received compression therapy alone. Her postoperative course was uneventful, without any kind of thrombosis. In the presence of FXII deficiency, ETA could be safely performed.

## Introduction

Factor XII (FXII) deficiency is a rare condition of the coagulation system with autosomal-recessive inheritance.^1)^ Patients with FXII deficiency have shown lack of bleeding tendency, despite prolonged activated partial thromboplastin time (APTT).^2)^ However, several reports in the literature demonstrated an increased tendency toward thrombosis, although the relationship between FXII deficiency and prothrombotic condition remains controversial.^3–5)^ Because FXII deficiency is very rare, there were less than 30 reported cases in which surgical procedures were performed in patients with FXII deficiency.^2)^ In the literature, heparin or warfarin was used for prophylaxis of thrombosis in a few cases, and in some, whole blood or plasma transfusion was given as substitution therapy. Here, we report the first case of a patient with FXII deficiency who underwent endovenous thermal ablation (ETA) for varicose vein due to the incompetent great saphenous vein (GSV). ETA was successfully carried out as a day surgery, with compression therapy alone as prophylaxis for thrombosis.

## Case Report

**Figure 1:**
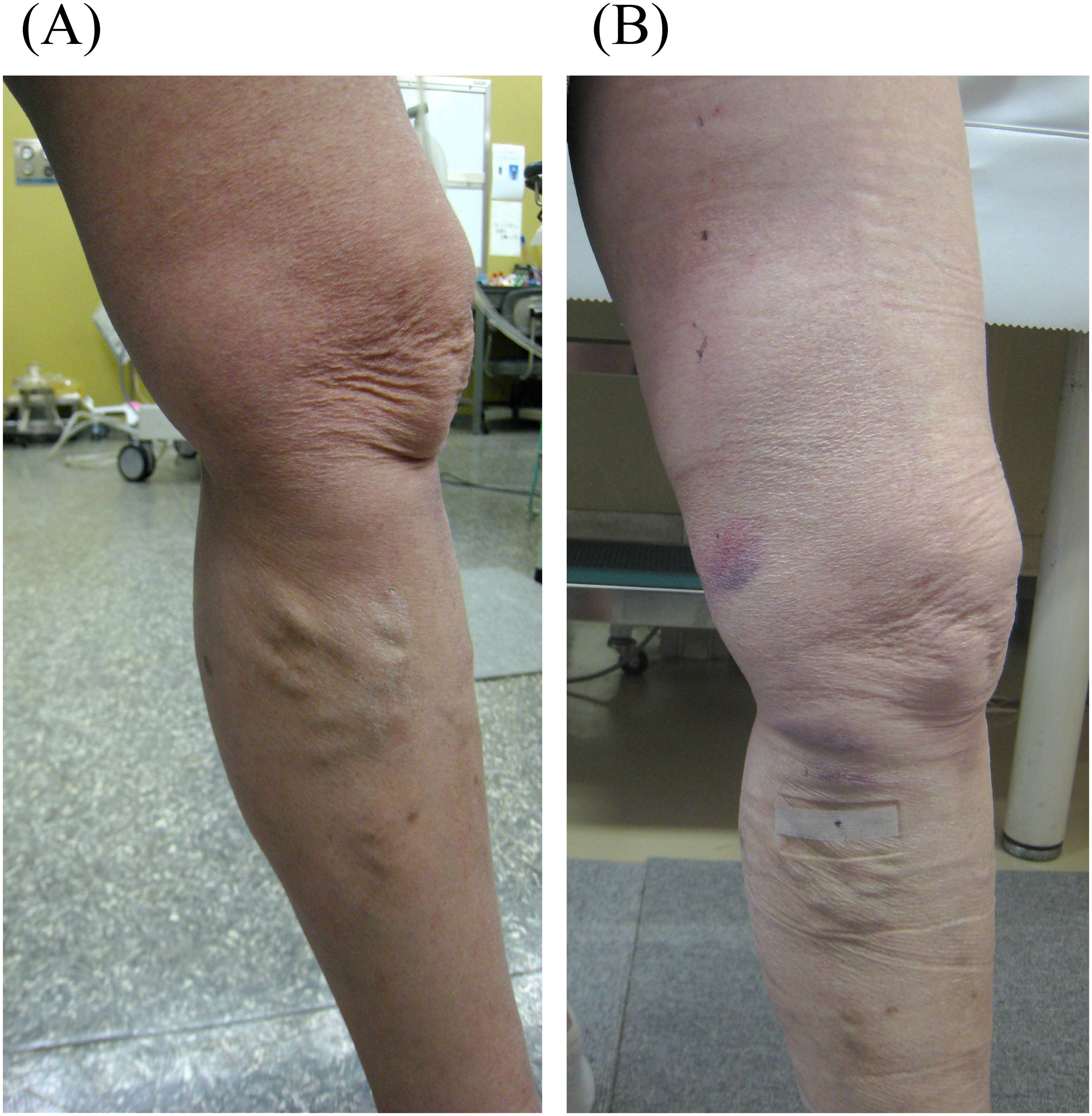
Fig. 1 (**A**) A 67-year-old woman represented varicose veins as well as edema and eczema due to the incompetent great saphenous vein (GSV) in her left leg. (**B**) One day after the varicose vein surgery (endovenous thermal ablation (ETA) for the incompetent GSV), no adverse events, except for the small ecchymosis at the site of tumescent local anesthesia, were observed.

A 67-year-old woman came to our varicose vein center complaining of varicosities as well as edema and eczema in her left lower leg. Upon duplex ultrasound (DUS), she was diagnosed of having primary varicose vein due to the incompetent GSV, with Clinical Etiological Anatomical Pathophysiological (CEAP) classification C2s C3s C4as Ep As P_R_ (**Fig. 1A**). DUS did not demonstrate thrombosis either in the deep veins or the superficial veins. She had no history of excessive bleeding or thrombosis on her conventional open appendectomy at the age of 15 or on her delivery of two children. There was no family history of bleeding tendency or thrombosis. Since she strongly desired to have varicose vein surgery for the cure of stasis edema and eczema, preoperative examination was performed, showing prolonged APTT to 180.0 seconds (**Table 1**). Both bleeding time and prothrombin time were within the normal range. To investigate the cause for abnormal APTT, she was referred to the department of hematology in a university hospital near our varicose vein center. The absence of medical history with bleeding tendency indicates that she does not have hemophilia (congenital or acquired) or von Willebrand disease. Further blood examinations demonstrated that FXII activity was 1% (normal range, 60%–140%) (**Table 1**). Other coagulation parameters were within the normal range. Based on these results, she was diagnosed of having severe (less or equal to 1% of normal) FXII deficiency. Our varicose vein center did not have any experience of surgery for patients with FXII deficiency. In addition, through literature search in PubMed, no reports are available on varicose vein surgery for patients with FXII deficiency. On the other hand, her attending hematologist approved for her to undergo varicose vein surgery without any treatment for FXII deficiency. Moreover, she had experienced conventional open appendectomy and vaginal delivery without any hemorrhagic or thrombotic complications. Taken together, because she strongly desired to have surgery for the varicose veins, we planned ETA for the incompetent GSV. Under local anesthesia combined with tumescent local anesthesia (TLA), ETA was performed with the use of a 1470-nm diode laser (ELVeS 1470, Integral Corp., Tokyo, Japan) and radial two-ring fiber (ELVeS Radial 2ring™ fiber, Integral Corp.). The ETA procedure was based on the reports of others.^6)^ The optical fiber was inserted at the distal side of the GSV through a 6-Fr sheath by DUS guidance. After TLA, from the point 2 cm distal from the saphenofemoral junction, the GSV in the thigh portion was ablated. The length of the ablated GSV was 27 cm, and the average linear endovenous energy density was 81.1 J/cm. We did not perform concomitant phlebectomy for the varicosities in the calf. Immediately after the procedure, a compression bandage was applied to the thigh and calf. These procedures were performed as a day surgery. She was followed up on an outpatient basis, and DUS was performed 1 day, 1 week, 1 month, 3 months, 6 months, and 1 year after the operation. Either deep or superficial vein thrombosis was not found postoperatively. On the first postoperative day (POD), DUS showed class 1 endovenous heat-induced thrombus (EHIT), which disappeared thereafter. Her postoperative course was uneventful, except for the tiny ecchymosis in the TLA area on the first POD (**Fig. 1B**).

**Table 1:** Table 1 Laboratory analysis on coagulation system

Laboratory parameters		Normal range
Platelet count	29.4	15.8–34.8 (×10^4^/μL)
Bleeding time	2	2–5 (min)
Prothrombin time	12.7	0–14 (sec)
INR	0.95	
APTT	180	24.5–33.8 (sec)
Fibrinogen	289	200–400 (mg/dL)
D-dimer	0.5	0–0.9 (μ/mL)
Factor XI activity	105	60–140 (%)
Factor XII activity	1	60–140 (%)
Lupus anticoagulant	0.99	0–1.29
Anti-cardiolipin beta 2 GPI Ab	2.2	0–3.4 (U/mL)
Anti-thrombin III activity	116	81–123 (%)
Protein C activity	143	64–135 (%)
Protein S activity	104	60–127 (%)

INR: international normalized ratio; APTT: activated partial thromboplastin time; GPI: glycoprotein I; Ab: antibody

## Discussion

In 1955, Ratnoff et al. first reported a case with FXII deficiency.^7)^ In the case, John Hageman and his two sisters had prolonged whole blood clotting time without abnormal bleeding history. The deficiency was found to be autosomal-recessive, and FXII was initially called Hageman factor.

The problems caused by FXII deficiency have been controversial, since it is a rare condition.^1)^ With the lack of bleeding tendency, FXII deficiency was originally suspected to be the risk for thrombosis, because John Hageman died from pulmonary embolism.^7)^ However, this was not the case because his pulmonary embolism had resulted from immobilization after hip fracture, which was presumably an independent risk factor for venous thromboembolism (VTE). Later, several reports described venous thrombosis in patients with FXII deficiency.^3–5)^ However, Koster et al. reported that there might be no relationship between FXII deficiency and increased risk for thrombosis.^8)^ They conducted a case control study to clarify the relationship between factor XII deficiency and venous thrombosis and revealed that there was no increase in the prevalence of FXII deficiency among patients with a first episode of venous thrombosis. Finally, Girolami’s group investigated the incidence of thrombotic events in patients with severe FXII deficiency, in comparison with the unaffected family members as controls.^1)^ They found that the incidence was similar in patients with FXII deficiency and in the unaffected members, indicating that FXII deficiency is not associated with an increased risk for venous thrombosis.

Because FXII deficiency is a rare condition, less than 30 cases of surgery have been reported in patients with severe FXII deficiency.^2)^ The cases contained major surgical procedures, including cholecystectomies, mastectomies, and a few cases of cardiac surgery. Despite the increasing evidence that FXII deficiency is not associated with excessive bleeding or thrombosis, preoperative plasma administration or prophylactic heparin was given in a few cases.

ETA is recommended as the first-choice procedure for symptomatic varicose veins.^9)^ Since the therapeutic goal of ETA is the obliteration of incompetent saphenous veins by thermal injury to the venous wall, ETA could be associated with concerns regarding the potential risks for VTE. Japanese nationwide survey found that venous thrombotic events, including VTE (deep vein thrombosis (DVT), pulmonary thromboembolism (PTE), and EHIT class 3–4), occurred in 0.194% of 43,203 cases.^10)^ Thrombotic complications should be avoided in ETA procedures, although they are infrequent. Usually, in our varicose vein center, for prophylaxis of perioperative thrombotic complications, we perform ETA as day surgery under local anesthesia with TLA, permitting an early ambulation immediately after the surgery. In addition, we apply post-procedural compression using elastic bandage or elastic stockings. In the case, the perioperative management was the same as usual, without the administration of antithrombotic agents such as heparin or direct oral anticoagulant, due to the following reasons. First, her attending hematologist approved for her to undergo varicose vein surgery without any substitution therapies or prophylaxis for thrombosis. Second, the results from the observational studies on FXII deficiency suggest that FXII deficiency was not associated with an increased risk for thrombosis. To be safe, concomitant phlebectomy by stab avulsion technique was not performed to shorten the operation time. Consequently, she has not experienced venous thrombotic complications (DVT, PTE, and EHIT class 3–4) in her perioperative period.

## Conclusion

Here, we described the first reported case of ETA surgery in a varicose vein patient with severe FXII deficiency. In the presence of severe FXII deficiency, ETA could be safely performed and the postoperative course was uneventful. Our observations, together with recent evidences from observational studies on FXII deficiency,^1,8)^ suggest that the incidence of thrombotic complications after ETA could be similar in patients with FXII deficiency to that in individuals with normal coagulation system. It seems that, in patients with FXII deficiency, perioperative management of ETA could be the same as usual, such as early ambulation with compression therapy.
